# Learning curve of ovarian cystectomy by vaginal natural orifice transluminal endoscopic surgery: a cumulative sum analysis

**DOI:** 10.3389/fmed.2024.1449446

**Published:** 2024-08-05

**Authors:** Kailiang Tan, Liufei Wei, Zengmei Deng, Desheng Yao, Li Jiang

**Affiliations:** ^1^Department of Gynecological Oncology, Guangxi Medical University Cancer Hospital, Nanning, Guangxi, China; ^2^Department of Obstetrics and Gynecology, Maternal and Child Health Hospital of Guangxi Zhuang Autonomous Region, Nanning, China

**Keywords:** cumulative sum analysis, endoscopy, learning curve, ovarian cystectomy, vNOTES

## Abstract

**Purpose:**

To identify the learning curve in ovarian cystectomy by vaginal natural orifice transluminal endoscopic surgery.

**Methods:**

Data consist of consecutively ordered patients who underwent ovarian cystectomy via vaginal natural orifice transluminal endoscopic surgery between May 2020 and June 2023. The learning curve of ovarian cystectomy via vaginal natural orifice transluminal endoscopic surgery was measured in terms of the operating time adjusted by multivariate linear regression. A cumulative sum analysis was performed to establish the learning curve. Patients’ characteristics and surgical outcomes were compared based on the inflection points of this curve.

**Results:**

The learning curve was divided into two unique phases: phase 1 (1–26 patients), and phase 2 (27–40 patients). The expected operating time in phase 2 was shorter than in phase 1 (86.4 ± 11.2 min vs. 102.0 ± 22.7 min, *p* = 0.021). The time to first postoperative flatus was shorter in phase 2 compared with phase 1 (14.6 ± 6.5 h vs. 20.6 ± 6.3 h, respectively, *p* = 0.008). No significant differences were observed in terms of patient’s age, BMI, tumor size, parity, bilateral ovarian tumor, pathological diagnoses, estimated blood loss, postoperative pain score, or perioperative complications between the two phases.

**Conclusion:**

Proficiency in ovarian cystectomy by vaginal natural orifice transluminal endoscopic surgery was achieved after 26 surgeries based on cumulative sum analysis. These findings may provide insight for structured training programs of ovarian cystectomy via vaginal natural orifice transluminal endoscopic surgery.

## Introduction

Benign ovarian cysts are one of the most common gynecological disorders, accounting for 6.6% in reproductive age (1). Some of these patients require a surgical procedure (2). Ovarian cystectomy is the primary surgical procedure for these patients who desire continued fertility (3). Minimally invasive surgery is becoming increasingly popular owing to benefits reflected in pain reduction, rapid recovery, and minimal cosmetic impacts (4, 5). A novel minimally invasive surgery, vaginal natural orifice transluminal endoscopic surgery (vNOTES), conducted through a colpotomy without abdominal incisions, enhances the above advantages (6–10). vNOTES has been applied to hysterectomy, oophorectomy, and ovarian cystectomy for patients with benign gynecological disorders (7, 11–14). vNOTES ovarian cystectomy was first reportedly performed on four patients in 2012 (6). Afterwards, ovarian cystectomy via vNOTES has been gradually implemented (7, 8, 13).

Nevertheless, vNOTES ovarian cystectomy is still challenging for experienced gynecological laparoscopists because surgeons must adapt to several difficulties such as limited surgical space, anatomically reverse direction, and mutual interference of surgical instruments (6, 8, 15). It is useful to evaluate the learning curve of ovarian cystectomy by vNOTES for purposes of surgical training, for this has not previously been undertaken. The objective of the current study is to establish and analyze the learning curve for vNOTES ovarian cystectomy based on an experienced gynecological laparoscopist using cumulative sum (CUSUM) methodology.

## Materials and methods

All data are from consecutively ordered patients who underwent ovarian cystectomy by vaginal natural orifice transluminal endoscopic surgery (vNOTES). These surgeries were conducted by an experienced laparoscopist between May 2020 and June 2023. Data were collected from a retrospective database of the Maternal and Child Health Hospital of Guangxi Zhuang Autonomous Region. The participating surgeon’s experience included more than 2000 cases of conventional laparoscopic surgeries and more than 30 cases of ovarian cystectomy via tranumbilical laparoendoscopic single-site surgery (LEES). The inclusion criteria for this study are as follows: (1) Women between 18 and 45 years of age; (2) Patients diagnosed with benign ovarian mass based on either ultrasound examination or nuclear magnetic resonance (MRI) preoperatively; and (3) Patients received vNOTES ovarian cystectomy as the primary treatment. Patients who did not undergo vNOTES ovarian cystectomy were excluded from this study. Because the main objective of the study is the surgeon’s performance, conversion related to adhesions was also excluded as described in a previous report (16). This study was approved by the Institutional Review Board ([2023] 11-2).

Preoperative variables, including baseline characteristics such as patients’ age, BMI, sexual history, parity, and tumor size, were collected. The surgical outcomes comprised operating time, estimated blood loss, postoperative pain score, time to first postoperative flatus, pathological diagnoses, and perioperative complications. Complications were defined by Clavien Dindo classification grade (17). Tumor size was measured as the average of length, width, and height of the ovarian tumor based on the findings of transvaginal ultrasound. When patients had bilateral ovarian tumors, the larger values of tumor size were selected for analysis.

### Surgical procedures

The patient was positioned in dorsal lithotomy and Trendelenburg position under general endotracheal anesthesia. Prophylactic antibiotics (0.5 g of metronidazole and 1.5 g of Cefuroxime) were administered 30 min prior to incision. Disinfecting and draping were then administered. Vaginal retractors were inserted to expose the cervix and vagina which were then sterilized with povidone-iodine. The posterior fornix of the vagina was completely exposed via pulling the cervix forward and downward. A 2.5 cm to 3 cm incision of posterior colpotomy was performed using cold scissors. Then the peritoneum was opened to access the peritoneal cavity. A laparoscopic single-site Platform (HangTian KaDi Technology R&D Institute, Beijing, China) was used as a vNOTES port (18). A wet gauze was placed in the peritoneal cavity to keep the intestine clear of the field of operation. A pneumoperitoneum was produced with the pressure of 10 mmHg. Conventional laparoscopic instruments were used for the surgery, which included a 0-degree telescope, monopolar, bipolar, cold scissor, and needle holder. The posterior pelvic cavity was probed, and the uterus, bilateral adnexa, and ovarian tumor were identified. A cortical incision was made using a monopolar electrosurgery and a cleavage plane was identified. Ovarian cystectomy was then performed using grasping forceps and cold scissors. Incidental hemorrhaging was coagulated using electrosurgical bipolar. The ovarian remnants were sutured for hemostasis using 2–0 polyglycolic acid suture in cases of excessive bleeding. The wet gauze was then withdrawn and the colpotomy incision was closed with 0–0 polyglycolic acid suture (Supplementary Figures S1–S8).

### Statistical analysis

Categorical data are presented as frequencies and percentages. Normally distributed data and skewed data are described by mean ± SD and median (25th, 75th), respectively. Categorical variables were compared using either the Chi-squared test or Fisher’s Exact test. Independent sample-*t* tests and Mann–Whitney tests were used to compare the normally distributed data and skewed data, respectively. Statistical significance was set at a two-sided *p* value of <0.05. Statistical analyses were performed using the SPSS version 26.0 software.

### Learning curve analysis

The learning curve of the vNOTES ovarian cystectomy was measured using the operating time over the course of the study. A multivariate linear regression was performed to evaluate the factors affecting the operating time (OT). An expected operating time (EOT) for each patient was calculated based on the multivariate linear regression equation. A cumulative sum (CUSUM) analysis was performed to analyze the learning curve. Patients were arranged chronologically from the earliest to the latest date of surgery. The CUSUM of the first patient was the difference between EOT for the first patient and the mean EOT for all patients. The CUSUM of the second patient was CUSUM of the previous patient plus the difference between EOT for the second patient and the mean EOT of all patients. This recursive process continued until the CUSUM of the last patient was calculated as zero.

## Results

Forty cases that underwent ovarian cystectomy by vNOTES between May 2020 and June 2023 were included in this study. Patient demographics, surgical characteristics and pathological diagnoses are summarized in Table 1. Only one case (2.5%) was complicated with postoperative uroschesis. Parity, bilateral ovarian tumor, and tumor size were found to be factors impacting operating time based on a multivariate linear regression analysis (Table 2). An expected operating time (EOT) for each patient was calculated according to the linear regression equation (EOT = 52.161 + 42.223 × Bilateral ovarian tumor +5.467 × Tumor size – 19.267 × Parity).

**Table 1:** Overall patient baseline demographic and surgical details.

Variables	Categories/unit	Mean ± SD/median (IQR)/*n* (%)
Age	years	29.9 ± 4.9
BMI	kg/m^2^	21.0 ± 2.4
Tumor size	cm	5.1 ± 1.4
Parity	Yes/No	27 (67.5)/13 (32.5)
Bilateral ovarian tumor	Yes/No	6 (15)/34 (85)
Operating time	min	96.5 ± 29.5
Estimated Blood loss	ml	30 (20, 50)
Time to first flatus	h	18.5 ± 6.9
Postoperative pain score	–	1 (0, 1)
Pathological diagnoses		
Dermoid cyst	Yes/No	37 (92.5)/3 (7.5)
Mucinous cystadenoma	Yes/No	1 (2.5)/39 (97.5)
Serous cystadenoma	Yes/No	1 (2.5)/39 (97.5)
Simple cyst	Yes/No	1 (2.5)/39 (97.5)

SD, standard deviation; IQR, interquartile range; BMI, body mass index.

**Table 2:** Multivariate linear regression analysis of the effects of operative time.

Variables	Unstandardized coefficients		Standardized coefficients	*t*	*p*-value	95%CI
	B	Std. error	Beta			
Constant	52.161	19.759		2.64	0.012	12.089–92.234
Parity	−19.267	7.584	−0.310	−2.54	0.016	−34.648--3.885
Bilateral ovarian tumor	42.223	10.384	0.517	4.066	<0.001	21.165–63.282
Tumor size	5.467	2.609	0.265	2.096	0.043	0.177–10.758

B, partial regression coefficient; Std, standard; CI, confidence interval.

**Figure 1:**
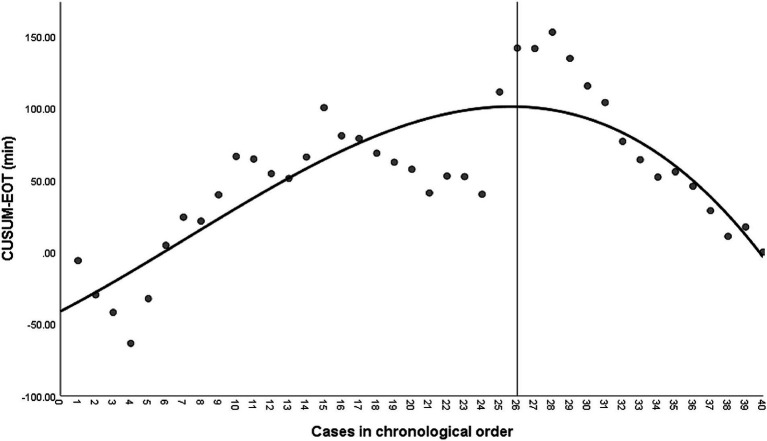
Cumulative sum (CUSUM) of expected operating time (EOT) plotted against a case number. The black line represents the curve of best fit for the plot (a cubic model equation CUSUM-EOT = −41.348 + 6.333*patient number + 0.156*patient number2–0.007* patient number3; R2 = 0.696).

The CUSUM-EOT learning curve was best modelled as a cubic model equation (CUSUM-EOT = −41.348 + 6.333 × patient number + 0.156 × patient number^2^–0.007× patient number^3^; *R*^2^ = 0.696) Figure 1. Analysis of the CUSUM-EOT learning curve revealed an inflection point at 26 procedures. The CUSUM-EOT learning curve comprised two unique phases: phase 1 (1–26 patients), phase 2 (27–40 patients). Comparisons of patient characteristics and surgical details were performed between these two phases (Table 3). There were no statistically significant differences in age, BMI, tumor size, parity, bilateral ovarian tumor, pathological diagnoses, estimated blood loss, postoperative pain score, or perioperative complications between the two groups. The EOT in phase 2 was shorter than in phase 1 (86.4 ± 11.2 min vs. 102.0 ± 22.7 min, *p* = 0.021). The time to first postoperative flatus was shorter in phase 2 compared with phase 1 (14.6 ± 6.5 h vs. 20.6 ± 6.3 h, respectively, *p* = 0.008).

**Table 3:** Interphase comparisons of patient characteristics and surgical details.

Variables	Phase 1	Phase 2	*p*-value
Age (year)	29.5 ± 4.1	29.6 ± 6.1	0.912
BMI (kg/m^2^)	20.6 ± 2.3	21.8 ± 2.5	0.127
Tumor size (cm)	5.4 ± 1.6	4.6 ± 0.9	0.072
Parity			
Yes	17 (65.4%)	10 (71.4%)	0.972
No	9 (34.6%)	4 (28.6%)	
Bilateral ovarian tumor			
Yes	6 (23.1%)	0 (0.0%)	0.074
No	20 (76.9%)	14 (100%)	
Pathological diagnoses			
Dermoid cyst	24 (92.3%)	13 (92.9%)	1.000
Non-Dermoid cyst	2 (7.7%)	1 (7.1%)	
OT (min)	102.5 ± 27.9	85.5 ± 30.2	0.083
EOT (min)	102.0 ± 22.7	86.4 ± 11.2	**0.021**
Estimated blood loss, median, (IQR), ml	30 (20, 50)	30 (20,50)	0.524
Time to first flatus (h)	20.6 ± 6.3	14.6 ± 6.5	**0.008**
Postoperative pain score, median, (IQR)	1 (0, 1)	1 (0,1)	0.926
Perioperative complications			
Uroschesis^†^	1 (3.8%)	0 (0.0%)	1.000

IQR, interquartile range; BMI, body mass index; OT, operative time; EOT, expected operative time; †, uroschesis was defined as Grade I based on the Clavien Dindo classification grade. Bold values means statistical significance.

## Discussion

vNOTES, a novel minimally invasive surgery, has been garnering attention in the gynecological field owing to the advantages it offers, including elimination of incision scars on the abdomen, less postoperative pain and more rapid recovery (7, 12, 19). However, vNOTES ovarian cystectomy is still challenging for experienced gynecological laparoscopists. The aim of the current study is to establish the learning curve for vNOTES ovarian cystectomy using the cumulative sum (CUSUM) method. Based on the findings of this study, data were categorized in two unique groups confirming the learning curve effect on ovarian cystectomy by vNOTES: Phase 1 (26 initial patients) represents the skills acquisition; phase 2 (27–40 patients) represents the proficiency period.

A learning curve based on CUSUM analysis can identify the optimal format of structured training programs for surgical procedures (16). A prior report indicates that a well-trained gynecologic endoscopist can achieve surgical proficiency in transvaginal natural orifice for ovarian cystectomy after 36 cases (20). In the present study, proficiency was determined after 26 patients, indicating that the learning curve appears to be slightly shorter compared with previous research (20). The difference may be explained by the following reasons. Firstly, the major procedure of ovarian cystectomy was performed via conventional transvaginal surgery under direct vision in the prior study (20). In the present study, vNOTES ovarian cystectomy was performed using a laparoscopic instrument. Secondly, most of the ovarian tumors in the current study are Dermoid cysts (92.5%), which is more than in the previous study (64.7%) (20). The different proportion of the pathological diagnoses may shorten the learning curve to achieve proficiency.

The learning curve of the surgical procedure using CUSUM analysis illustrates the course of a surgeon’s proficiency in performing a surgery (16). The CUSUM analysis is also used to assess the learning curve of multiple surgeons and multiple centers (21). In this instance, a learning curve may represent the performance of a group rather than that of an individual surgeon (21). Intraoperative parameters such as blood loss and operating time, reflecting a surgeon’s competence in a surgical procedure, were typically used to assess the learning curves (20–22). Multiple factors may impact the operative time besides the surgeon’s surgical skills (21). In the current study, parity, bilateral ovarian tumor, and tumor size were found to impact the operating time according to the multivariate linear regression analysis. Therefore, the expected operating time (EOT) was calculated to adjust for the influence of the factors as previous research concluded (21). In the present study, the expected operative time (EOT) in phase 2 was shorter than in phase 1 (86.4 ± 11.2 min vs. 102.0 ± 22.7 min, *p* = 0.021), which is in alignment with previous reports (16, 20). Time to first flatus is shorter in phase 2 than in phase 1. This indicates the intestine was less disturbed as the surgeon’s competence improved. Although more complicated cases (bilateral, larger cyst size) occurred in phase 1, the effects have been adjusted to mitigate bias using a multivariate linear regression method.

Besides the operating time, success rates and complications were also included in the CUSUM learning curve analysis (16, 21, 23). It is important to note that a CUSUM analysis of a procedure may indicate a change of the referral pattern rather than a surgical failure rate (16). Additionally, the operating time and complication rate may increase in relation to more complex cases (21, 22, 24). In the present study, only one case produced a complication of uroschesis, accounting for 2.5%. The low complication rate of vNOTES ovarian cystectomy is consistent with a previous report (8). As the occurrence of complications is relatively low, it is not applicable to the CUSUM analysis of the learning curve. Notably, it reveals that vNOTES may lead to an increased trend of bleeding complications compared with laparoscopy in a prior trial. Thus, it is necessary to exert careful efforts to achieve complete hemostasis in vNOTES procedures.

To the best of our knowledge, this is the first report of a learning curve of ovarian cystectomy by vNOTES. A multivariate linear regression was used to adjust the effects on the operating time to mitigate bias. Although the current study, which included a single surgeon, can provide better homogeneity, it may not reflect the performance of other surgeons in different hospitals. It should be noted that this was a retrospective study, and thus a further prospective multiple center trial is warranted to confirm these findings. Additionally, because many patients did not accept the vNOTES in the local area, the course of the study was relatively lengthy. It is undeniable that the long duration of study may impact the learning curve. Moreover, vNOTES cystectomy is difficult to perform in cases of intra-abdominal adhesions, especially for patients with endometriosis or prior surgery. It may limit the application of vNOTES in these circumstances. The retroperitoneal approach may facilitate the surgery in these cases (25). Finally, as the dermoid cyst has an elevated rate of occurrence in the present study, the findings may not be characteristic of vNOTES cystectomy for other types of ovarian cysts.

## Conclusion

Proficiency in ovarian cystectomy employing vNOTES was achieved after 26 cases based on the CUSUM analysis. These findings may provide reliable information for structured training programs of ovarian cystectomy by vNOTES.

## Data availability statement

The raw data supporting the conclusions of this article will be made available by the authors, without undue reservation.

## Ethics statement

The studies involving humans were approved by Maternal and Child Health Hospital of Guangxi Zhuang Autonomous Region, China. The studies were conducted in accordance with the local legislation and institutional requirements. The participants provided their written informed consent to participate in this study.

## Author contributions

KT: Writing – original draft, Methodology. LW: Data curation, Writing – original draft. ZD: Resources, Writing – original draft. DY: Writing – review & editing. LJ: Conceptualization, Writing – review & editing.

## Supplementary material

SUPPLEMENTARY FIGURE S1 The posterior fornix of the vagina was completely exposed via pulling the cervix forward and downward.

SUPPLEMENTARY FIGURE S2 A laparoscopic single-site Platform was used as a vNOTES port.

SUPPLEMENTARY FIGURE S3 The posterior pelvic cavity was probed, and the right ovarian mass were identified.

SUPPLEMENTARY FIGURES S4 and S5 A cortical incision was made using a monopolar and a cleavage plane was identified. Ovarian cystectomy was performed using grasping forceps and cold scissors.

SUPPLEMENTARY FIGURES S6 and S7 The ovarian remnants were sutured for hemostasis by using 2–0 polyglycolic acid suture.

SUPPLEMENTARY FIGURE S8 Enclosed the colpotomy incision with 0–0 polyglycolic acid suture.

## References

[1] Borgfeldt C Andolf E. Transvaginal sonographic ovarian findings in a random sample of women 25–40 years old. Ultrasound Obstet Gynecol. (1999) 13:345–50. doi: 10.1046/J.1469-0705.1999.13050345.X 10380300

[2] Hilger WS Magrina JF Magtibay PM. Laparoscopic management of the adnexal mass. Clin Obstet Gynecol. (2006) 49:535–48. doi: 10.1097/00003081-200609000-00013 16885660

[3] Alammari R Lightfoot M Hc H. Impact of cystectomy on ovarian reserve: review of the literature. J Minim Invasive Gynecol. (2017) 24:247–57. doi: 10.1016/j.jmig.2016.12.010 28089684

[4] Yuen PM Yu KM Yip SK Lau WC Rogers MS Chang A. A randomized prospective study of laparoscopy and laparotomy in the management of benign ovarian masses. Am J Obstet Gynecol. (1997) 177:109–14. doi: 10.1016/S0002-9378(97)70447-2, PMID: 9240592

[5] Nowak M Szpakowski M Malinowski A Maciołek-Blewniewska G Wilczyński JR Władziński J. Laparoscopy and laparotomy in the operative treatment of ovarian cysts. Ginekol Pol. (2000) 71:1173–8. PMID: 11082998

[6] Lee CL Wu KY Su H Ueng SH Yen CF. Transvaginal natural-orifice transluminal endoscopic surgery (notes) in adnexal procedures. J Minim Invasive Gynecol. (2012) 19:509–13. doi: 10.1016/j.jmig.2012.02.005, PMID: 22425142

[7] Baekelandt J. Transvaginal natural orifice transluminal endoscopic surgery: a new approach to ovarian cystectomy. Fertil Steril. (2018) 109:366. doi: 10.1016/j.fertnstert.2017.10.037, PMID: 29246560

[8] Wang CJ Wu PY Kuo HH Yu HT Huang CY Tseng HT. Natural orifice transluminal endoscopic surgery-assisted versus laparoscopic ovarian cystectomy (Naoc vs. Loc): a case-matched study. Surg Endosc. (2016) 30:1227–34. doi: 10.1007/s00464-015-4315-6, PMID: 26139483

[9] Li YC Ku FC Kuo HH Tseng HJ Wang CJ. Transvaginal endoscopic surgery-assisted versus conventional laparoscopic Adnexectomy (Tvea vs. Cla): a propensity-matched study and literature review. Taiwan J Obstet Gynecol. (2017) 56:336–41. doi: 10.1016/j.tjog.2017.04.013, PMID: 28600044

[10] Baekelandt J Kapurubandara S. Benign Gynaecological procedures by vaginal natural orifice transluminal endoscopic surgery (Vnotes): complication data from a series of 1000 patients. Eur J Obstet Gynecol Reprod Biol. (2021) 256:221–4. doi: 10.1016/j.ejogrb.2020.10.059, PMID: 33248377

[11] Karkia R Giacchino T Taylor J Ghaffar A Gupta A Kovoor E. Hysterectomy and Adenextomy via transvaginal natural orifice transluminal endoscopic surgery (Vnotes): a Uk perspective with a case series of 33 patients. Eur J Obstet Gynecol Reprod Biol. (2019) 242:29–32. doi: 10.1016/j.ejogrb.2019.08.023, PMID: 31539766

[12] Baekelandt J de Mulder PA le Roy I Mathieu C Laenen A Enzlin P. Adnexectomy by vaginal natural orifice transluminal endoscopic surgery versus laparoscopy: results of a first randomised controlled trial (notable trial). BJOG. (2021) 128:1782–91. doi: 10.1111/1471-0528.16838, PMID: 34246198

[13] Zhang Y Jia Y Dai X Wang F Gong Y. Transvaginal natural orifice transluminal endoscopic surgery-assisted versus Transumbilical Laparoendoscopic single-site ovarian cystectomy for ovarian mature cystic Teratoma. A randomized controlled trial. Ginekol Pol. (2023) 95:343–9. doi: 10.5603/Gpl.95422 38099663

[14] Kim MS Noh JJ Kim TJ. Hysterectomy and adnexal procedures by vaginal natural orifice transluminal endoscopic surgery (Vnh): initial findings from a Korean surgeon. Front Med (Lausanne). (2020) 7:583147. doi: 10.3389/Fmed.2020.583147, PMID: 33693007 PMC7937714

[15] Reddy H Kim SW Plewniak K. Applications of vaginal natural orifice transluminal endoscopic surgery (vNOTES) in gynecologic surgery. Curr Opin Obstet Gynecol. (2022) 34:220–6. doi: 10.1097/GCO.0000000000000799, PMID: 35895964

[16] van Zanten F Schraffordt Koops SE Pasker-de Jong PCM Lenters E Schreuder H. Learning curve of robot-assisted laparoscopic Sacrocolpo(recto)Pexy: a cumulative sum analysis. Am J Obstet Gynecol. (2019) 221:483.E1–483.E11. doi: 10.1016/j.ajog.2019.05.037, PMID: 31152711

[17] Clavien PA Barkun J de Oliveira ML Vauthey JN Dindo D Schulick RD. The Clavien-Dindo classification of surgical complications: five-year experience. Ann Surg. (2009) 250:187–96. doi: 10.1097/SLA.0b013e3181b13ca2 19638912

[18] Zhang J Dai Y Leng J Zhu L Lang J Sun D. Hysterectomy and bilateral Adnexectomy using transvaginal natural orifice transluminal endoscopic surgery: the role of multichannel abdominal port and vaginal support ring. J Obstet Gynaecol Res. (2021) 47:2521–8. doi: 10.1111/jog.14752, PMID: 33880852

[19] Ahn KH Song JY Kim SH Lee KW Kim T. Transvaginal single-port natural orifice transluminal endoscopic surgery for benign uterine adnexal pathologies. J Minim Invasive Gynecol. (2012) 19:631–5. doi: 10.1016/J.Jmig.2012.04.001, PMID: 22763314

[20] Huang YT Yang LY Pan YB Huang HY Wu KY Wang CJ. Learning curve analysis of transvaginal natural orifice adnexal surgery. J Minim Invasive Gynecol. (2020) 27:489–97. doi: 10.1016/j.jmig.2019.04.009, PMID: 30980993

[21] van Ramshorst TME Edwin B Han H-S Nakamura M Yoon Y-S Ohtsuka T. Learning curves in laparoscopic distal pancreatectomy: a different experience for each generation. Int J Surg. (2023) 109:1648–55. doi: 10.1097/JS9.0000000000000408, PMID: 37144678 PMC10389345

[22] Nomi T Fuks D Kawaguchi Y Mal F Nakajima Y Gayet B. Learning curve for laparoscopic major hepatectomy. Br J Surg. (2015) 102:796–804. doi: 10.1002/bjs.9798 25873161

[23] Pp T Aj S Cp D Vw F. Evaluation of the learning curve in laparoscopic colorectal surgery: comparison of right-sided and left-sided resections. Ann Surg. (2005) 242:83–91. doi: 10.1097/01.sla.0000167857.14690.68 15973105 PMC1357708

[24] Wang CJ Go J Huang HY Wu KY Huang YT Liu YC. Learning curve analysis of transvaginal natural orifice transluminal endoscopic hysterectomy. BMC Surg. (2019) 19:88. doi: 10.1186/s12893-019-0554-0, PMID: 31291917 PMC6621966

[25] Comba C Demirayak G Simsek C Atas BS Özdemir İA. Transvaginal natural orifice transluminal endoscopic surgery (Vnotes) Total retroperitoneal sentinel lymph node biopsy for an endometrial Cancer patient with prior Colon Cancer surgery. Int J Gynecol Cancer. (2021) 31:1386–7. doi: 10.1136/Ijgc-2021-002710, PMID: 34380707

