# Supplementary figures and images for: Learning curve of ovarian cystectomy by vaginal natural orifice transluminal endoscopic surgery: a cumulative sum analysis
Source: Front Med (Lausanne). 2024 Aug 5;11:1449446. doi: 10.3389/fmed.2024.1449446 (PMC11330758; doi:10.3389/fmed.2024.1449446)

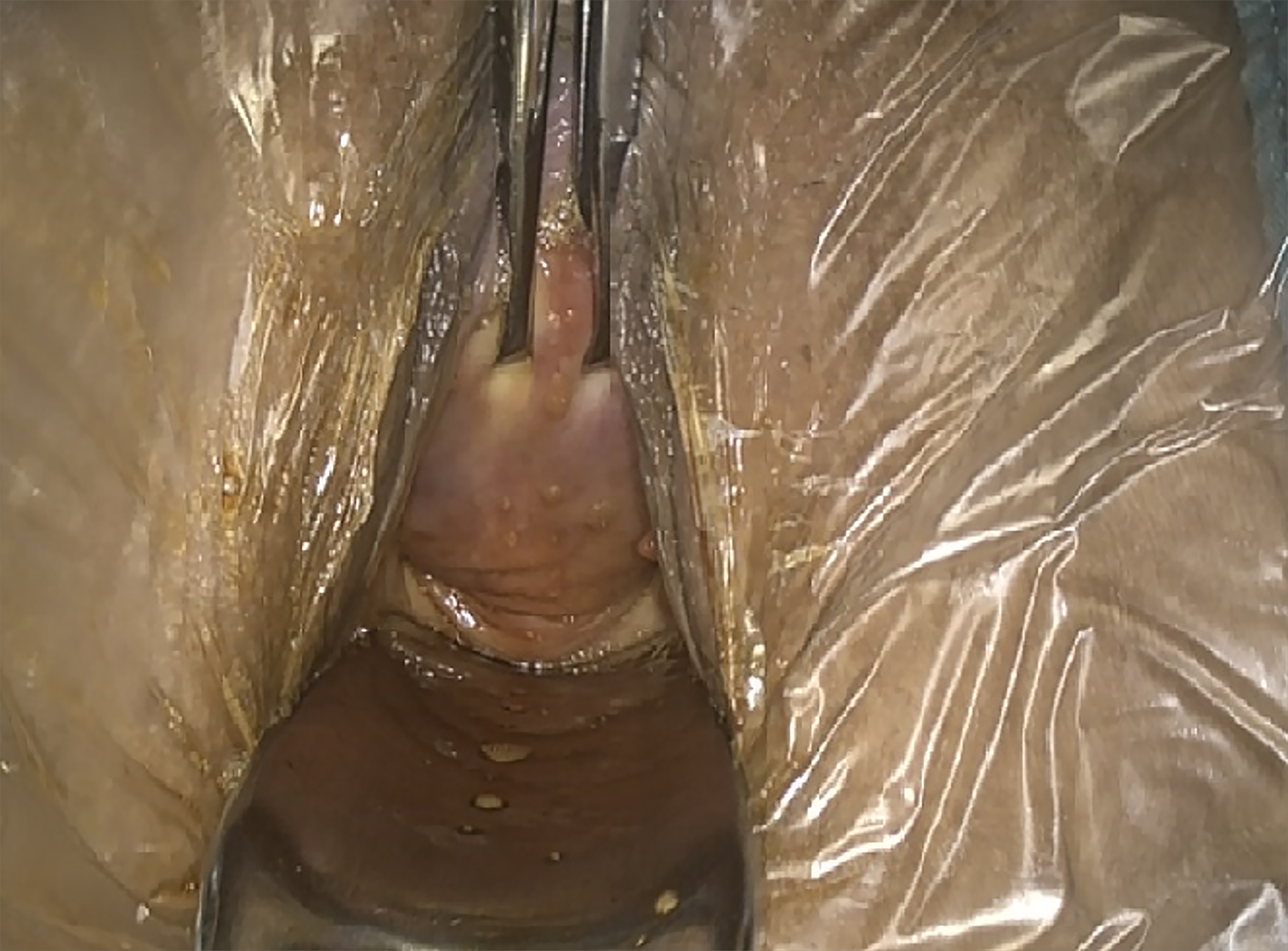

Supplement: SUPPLEMENTARY FIGURE S1 — The posterior fornix of the vagina was completely exposed via pulling the cervix forward and downward. [file Image_1.TIF]

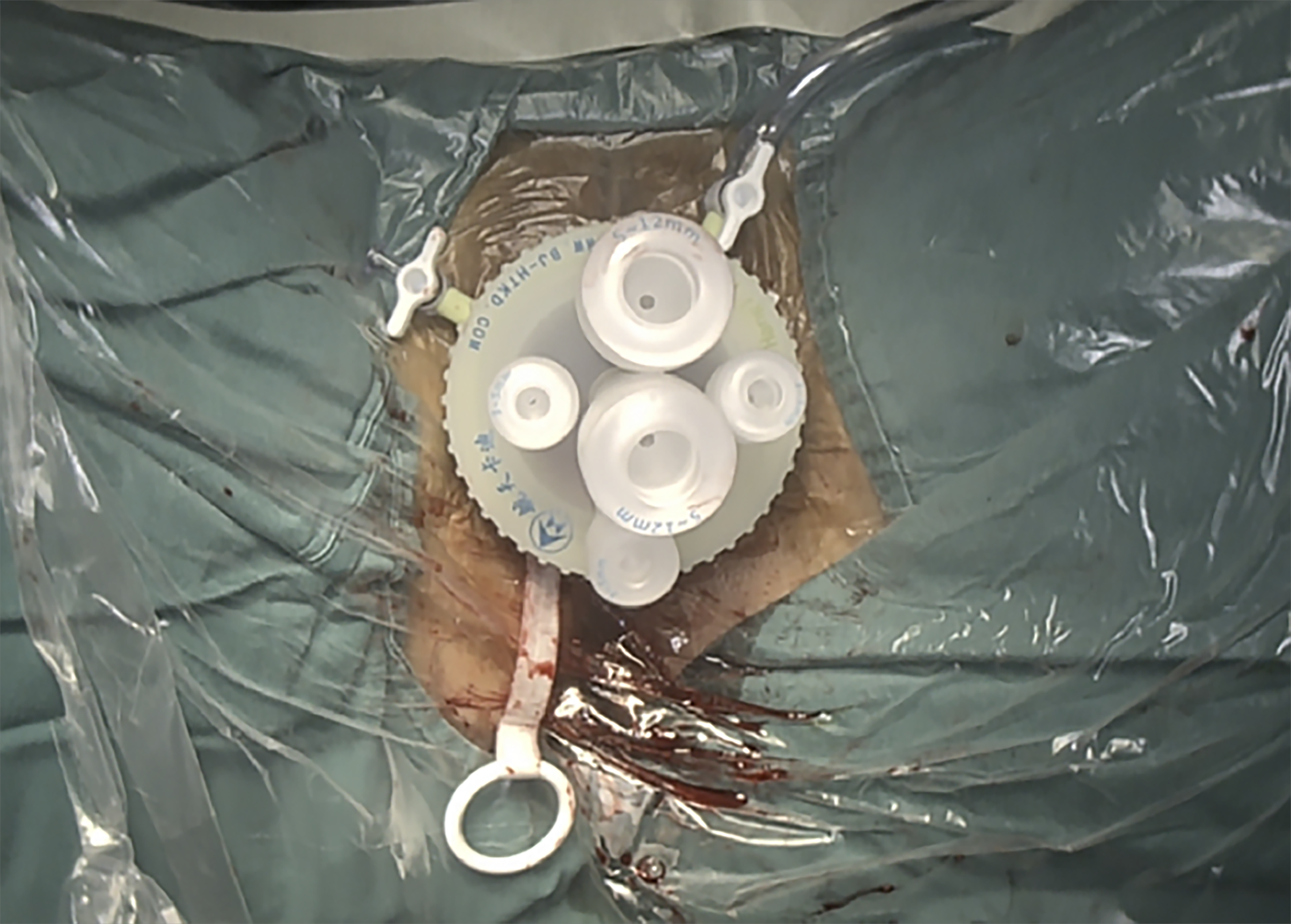

Supplement: SUPPLEMENTARY FIGURE S2 — A laparoscopic single-site Platform was used as a vNOTES port. [file Image_2.TIF]

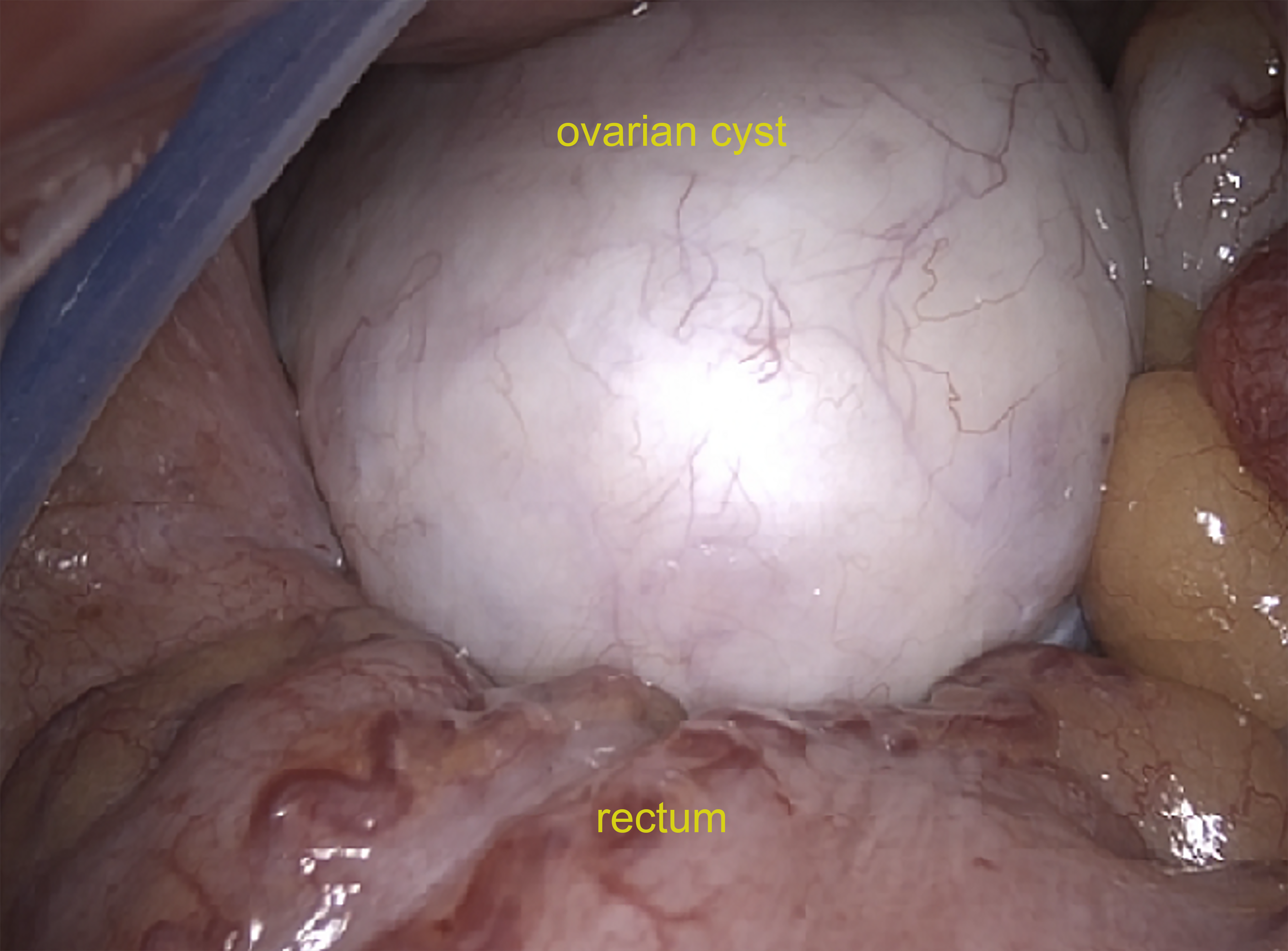

Supplement: SUPPLEMENTARY FIGURE S3 — The posterior pelvic cavity was probed, and the right ovarian mass were identified. [file Image_3.TIF]

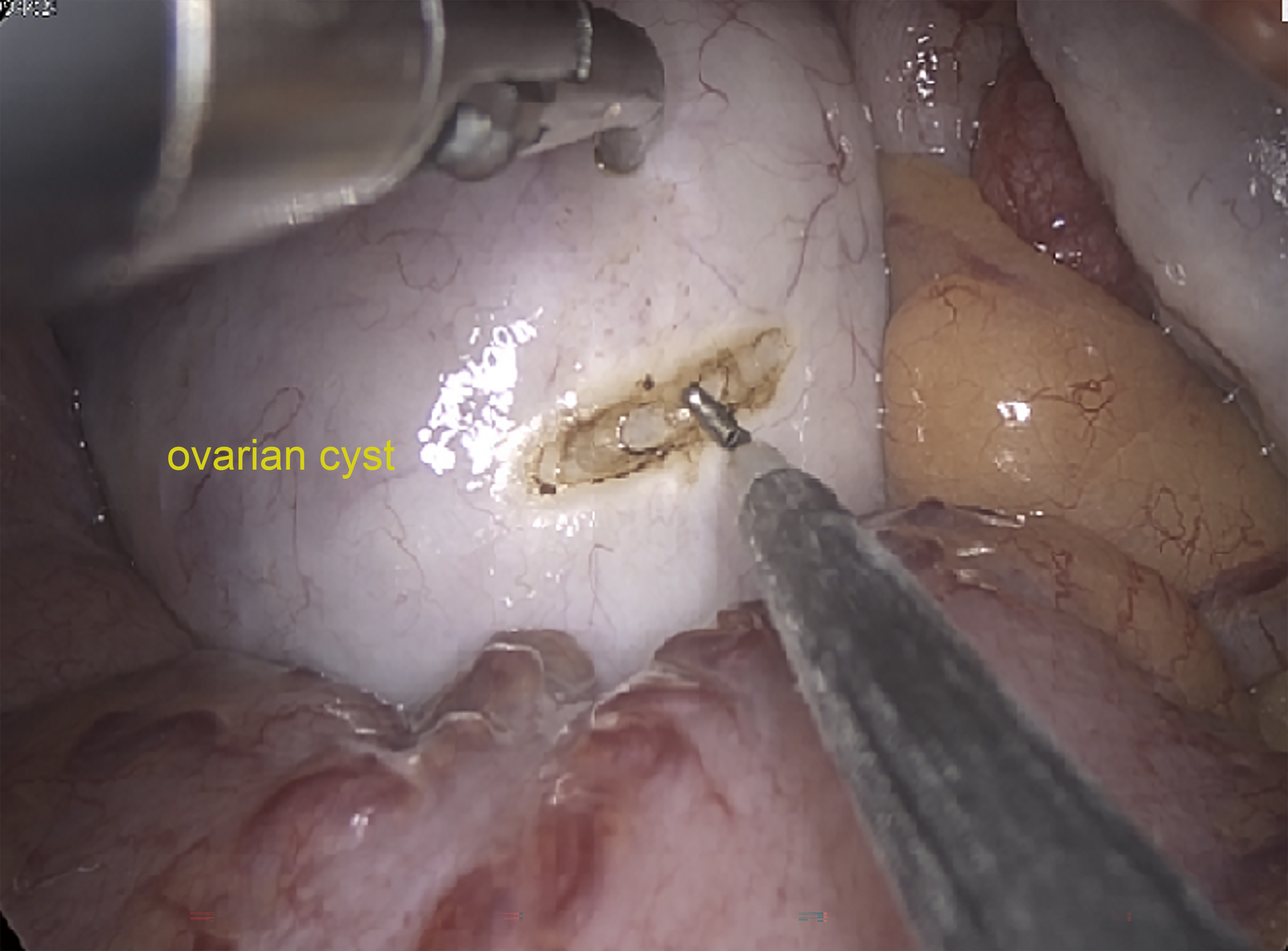

Supplement: SUPPLEMENTARY FIGURES S4 and S5 — A cortical incision was made using a monopolar and a cleavage plane was identified. Ovarian cystectomy was performed using grasping forceps and cold scissors. [file Image_4.TIF]

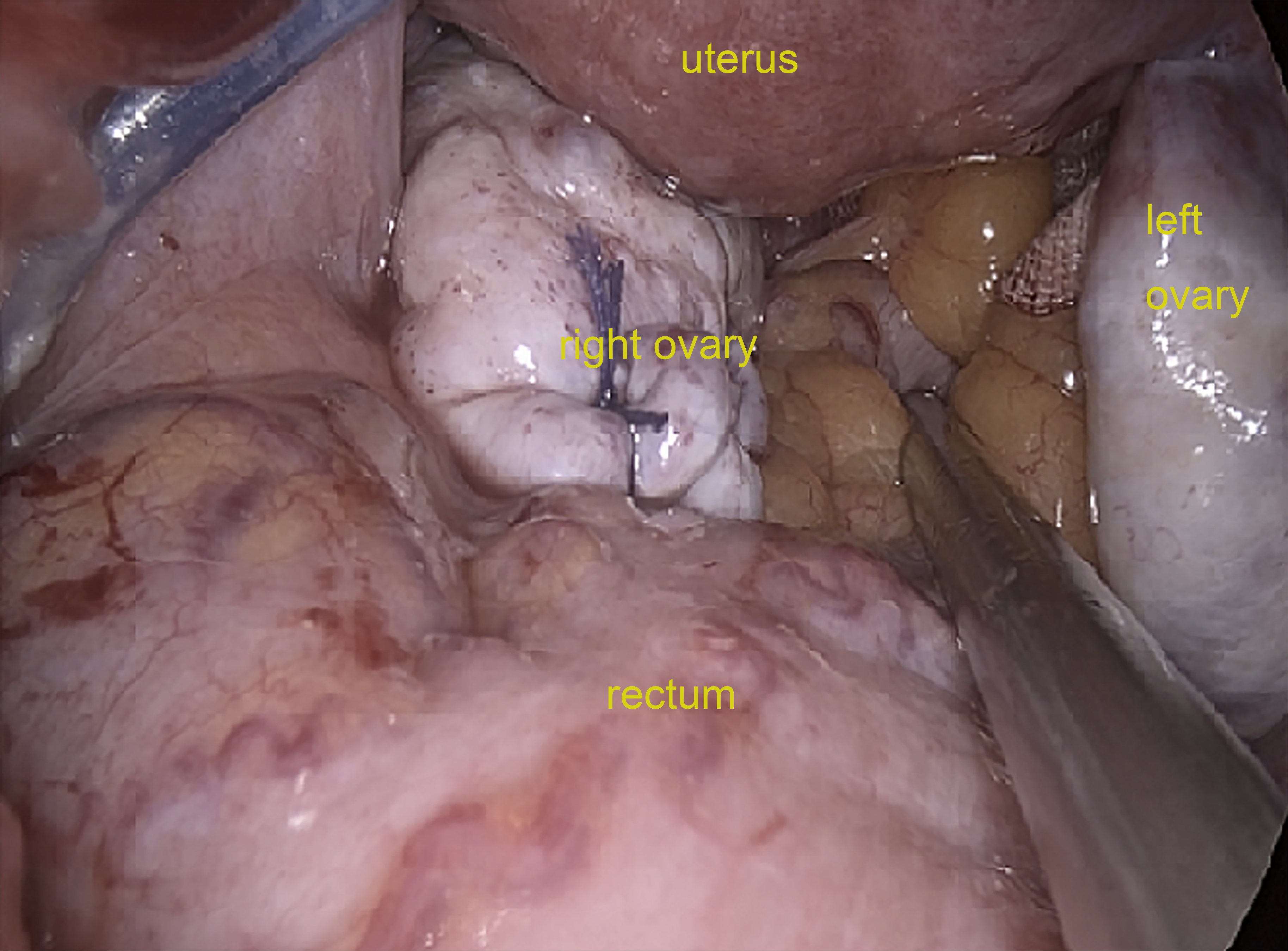

Supplement: SUPPLEMENTARY FIGURES S6 and S7 — The ovarian remnants were sutured for hemostasis by using 2–0 polyglycolic acid suture. [file Image_7.TIF]

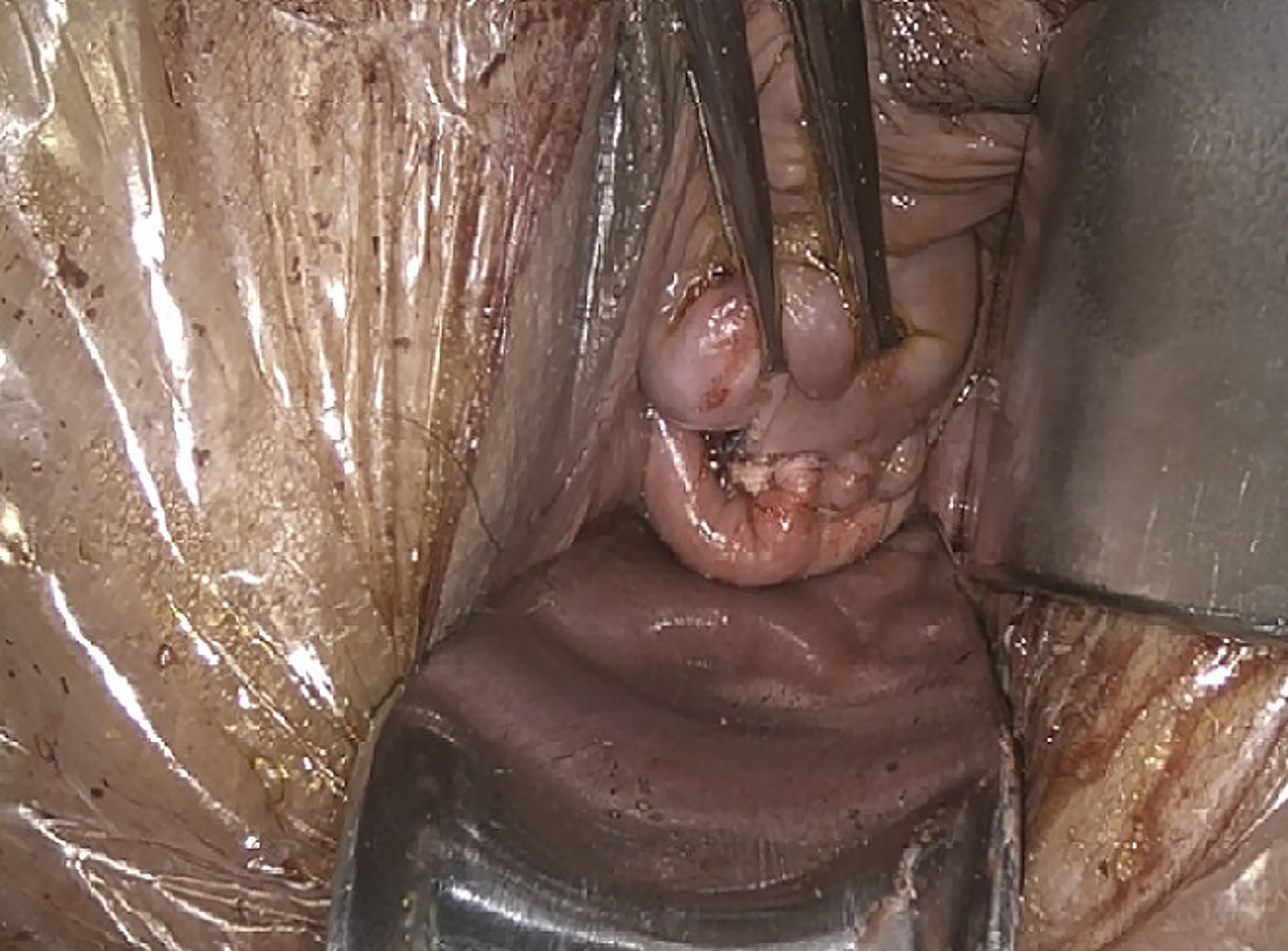

Supplement: SUPPLEMENTARY FIGURE S8 — Enclosed the colpotomy incision with 0–0 polyglycolic acid suture. [file Image_8.TIF]

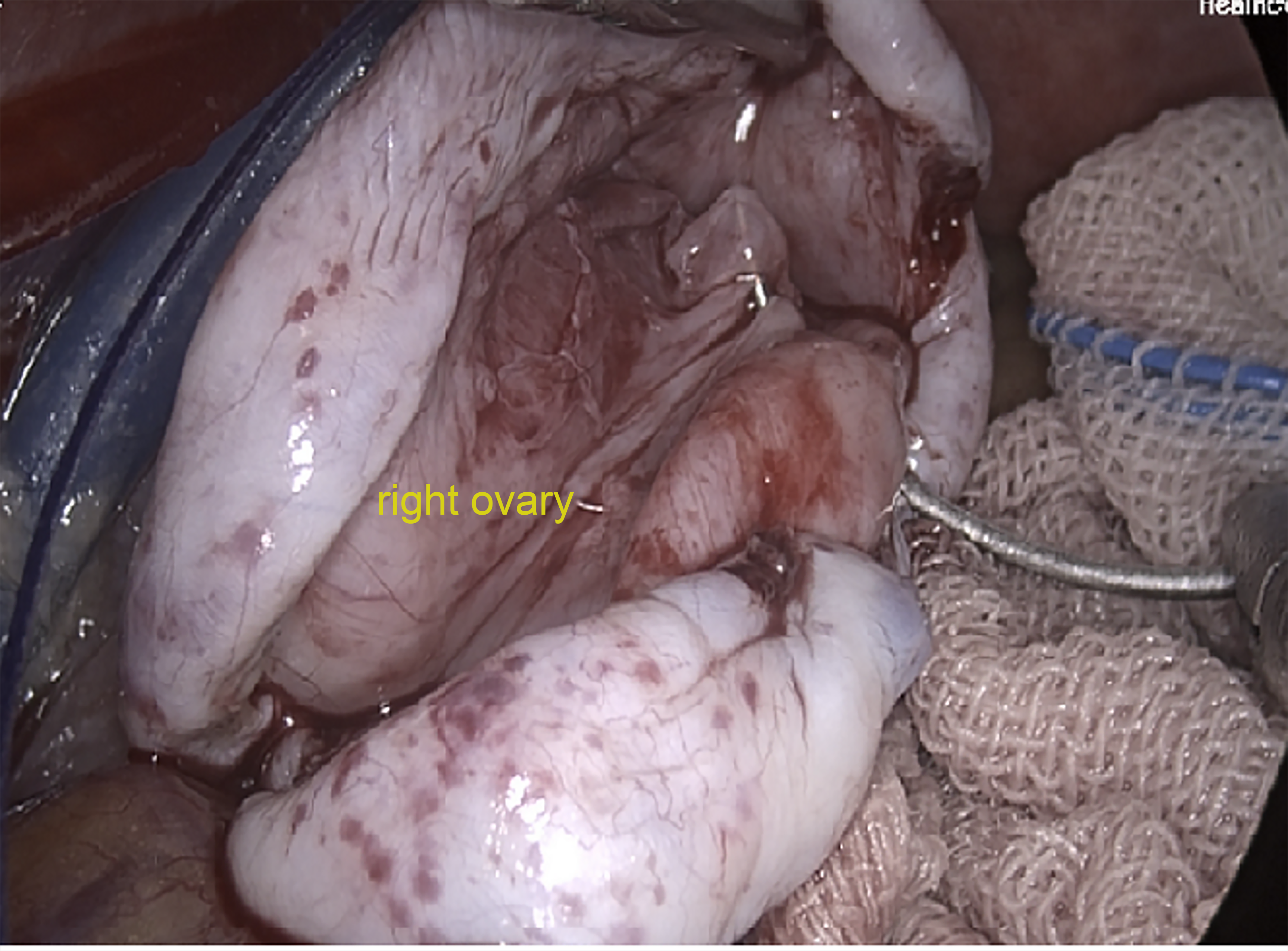

Supplement: Supplementary file 7 [file Image_6.TIF]

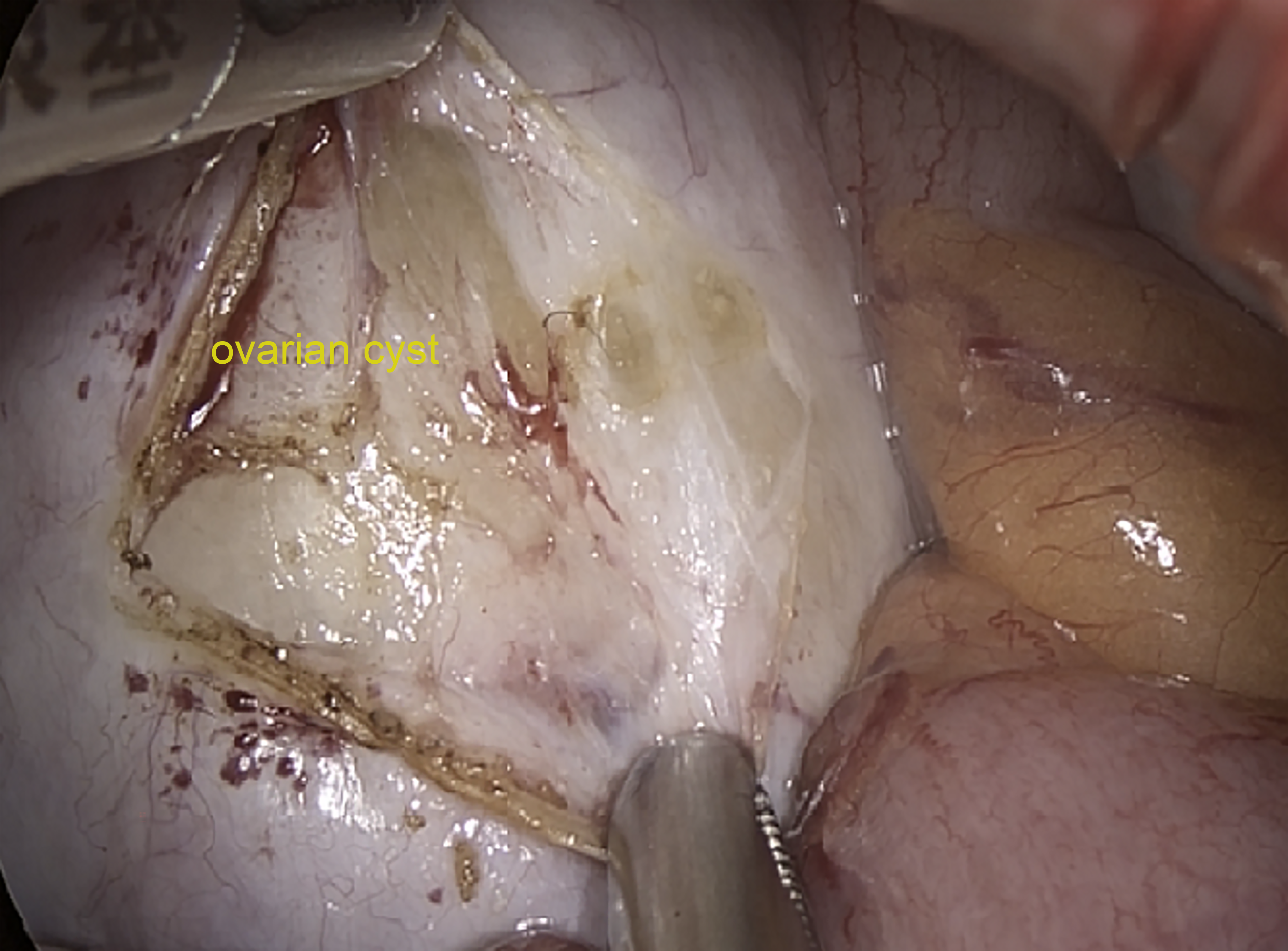

Supplement: Supplementary file 8 [file Image_5.TIF]
